# The PedS2/PedR2 Two-Component System Is Crucial for the Rare Earth Element Switch in Pseudomonas putida KT2440

**DOI:** 10.1128/mSphere.00376-18

**Published:** 2018-08-29

**Authors:** Matthias Wehrmann, Charlotte Berthelot, Patrick Billard, Janosch Klebensberger

**Affiliations:** a University of Stuttgart, Institute of Biochemistry and Technical Biochemistry, Stuttgart, Germany; b Université de Lorraine, LIEC UMR7360, Faculté des Sciences et Technologies, Vandoeuvre-lès-Nancy, France; c CNRS, LIEC UMR7360, Faculté des Sciences et Technologies, Vandoeuvre-lès-Nancy, France; University of Iowa

**Keywords:** lanthanides, LuxR-type regulator, PQQ, PedR2, PedS2, *Pseudomonas putida*, dehydrogenases, histidine kinase, periplasm, rare earth element switch, signal transduction, two-component regulatory systems

## Abstract

In Pseudomonas putida KT2440, two pyrroloquinoline quinone-dependent ethanol dehydrogenases (PQQ-EDHs) are responsible for the periplasmic oxidation of a broad variety of volatile organic compounds (VOCs). Depending on the availability of rare earth elements (REEs) of the lanthanide series (Ln^3+^), we have recently reported that the transcription of the genes encoding the Ca^2+^-utilizing enzyme PedE and the Ln^3+^-utilizing enzyme PedH are inversely regulated. With adaptive evolution experiments, site-specific mutations, transcriptional reporter fusions, and complementation approaches, we now demonstrate that the PedS2/PedR2 (PP_2671/PP_2672) two-component system (TCS) plays a central role in the observed REE-mediated switch of PQQ-EDHs in P. putida. We provide evidence that in the absence of lanthanum (La^3+^), the sensor histidine kinase PedS2 phosphorylates its cognate LuxR-type response regulator PedR2, which in turn not only activates *pedE* gene transcription but is also involved in repression of *pedH*. Our data further suggest that the presence of La^3+^ lowers kinase activity of PedS2, either by the direct binding of the metal ions to the periplasmic region of PedS2 or by an uncharacterized indirect interaction, leading to reduced levels of phosphorylated PedR2. Consequently, the decreasing *pedE* expression and concomitant alleviation of *pedH* repression causes—in conjunction with the transcriptional activation of the *pedH* gene by a yet unknown regulatory module—the Ln^3+^-dependent transition from PedE- to PedH-catalyzed oxidation of alcoholic VOCs.

**IMPORTANCE** The function of lanthanides for methanotrophic and methylotrophic bacteria is gaining increasing attention, while knowledge about the role of rare earth elements (REEs) in nonmethylotrophic bacteria is still limited. The present study investigates the recently described differential expression of the two PQQ-EDHs of P. putida in response to lanthanides. We demonstrate that a specific TCS is crucial for their inverse regulation and provide evidence for a dual regulatory function of the LuxR-type response regulator involved. Thus, our study represents the first detailed characterization of the molecular mechanism underlying the REE switch of PQQ-EDHs in a nonmethylotrophic bacterium and stimulates subsequent investigations for the identification of additional genes or phenotypic traits that might be coregulated during REE-dependent niche adaptation.

## Abstract (precis)

The function of lanthanides for methanotrophic and methylotrophic bacteria is gaining increasing attention, while knowledge about the role of rare earth elements (REEs) in nonmethylotrophic bacteria is still limited. The present study investigates the recently described differential expression of the two PQQ-EDHs of P. putida in response to lanthanides. We demonstrate that a specific TCS is crucial for their inverse regulation and provide evidence for a dual regulatory function of the LuxR-type response regulator involved. Thus, our study represents the first detailed characterization of the molecular mechanism underlying the REE switch of PQQ-EDHs in a nonmethylotrophic bacterium and stimulates subsequent investigations for the identification of additional genes or phenotypic traits that might be coregulated during REE-dependent niche adaptation.

## INTRODUCTION

In its natural soil habitat, Pseudomonas putida KT2440 is exposed to a broad range of potential carbon and energy sources (1–3), including plant-, fungus-, or bacterium-derived volatile organic compounds (VOCs) with alcohols or aldehydes as functional groups (4–6). For efficient capture and metabolism of such VOCs, P. putida makes use of two pyrroloquinoline quinone (PQQ)-dependent ethanol dehydrogenases (PQQ-EDHs)—namely, PedE and PedH—to carry out the initial oxidation steps in the periplasm of the cell (7, 8). In a recent study, we found that these two type I quinoproteins (9, 10) exhibit a similar substrate scope but require different metal cofactors (8). In contrast to PedE, which uses Ca^2+^ ions, PedH was characterized as a rare earth element (REE)-dependent enzyme that relies on the presence of lanthanides (Ln^3+^) for catalytic activity. Notably, due to their low solubility in most natural environments, REEs have long been considered to have no biological function (11). However, the discovery of the widespread occurrence of the REE-dependent XoxF type of PQQ-dependent methanol dehydrogenases (PQQ-MDHs), together with the more recent description of Ln^3+^-dependent PQQ-EDHs, has highlighted the important role of REEs for many bacterial species in various environmental compartments (8, 12–20).

While in the absence of Ln^3+^, the oxidation of methanol in methylotrophs is driven by Ca^2+^-dependent PQQ-MDHs (MxaF type), the presence of small amounts of REE ions is usually sufficient to trigger a transcriptional switch to the XoxF type of PQQ-MDHs. This inverse regulation, called the REE switch, has been reported for many methanotrophic and/or methylotrophic organisms (13, 16, 21–24). From the growing number of studies, it has become apparent that the molecular mechanism underlying this switch for PQQ-MDHs is complex and can substantially differ among species. For example, the inverse regulation in the nonmethanotrophic methylotroph Methylobacterium extorquens strain AM1 is controlled by two different two-component systems (TCSs) (MxcQE and MxbDM) and the orphan response regulator MxaB (25–27). In this organism, it has been found that the transcriptional activation of both enzymes, the Ca^2+^-dependent MxaF and the two Ln^3+^-dependent XoxF1 and XoxF2, is entirely lost in a Δ*xoxF1* Δ*xoxF2* double mutant (28). As a consequence, a complex regulation in which the different binding affinities of the *apo* form (no Ln^3+^ bound to the enzyme) and *holo* form (Ln^3+^ bound to the enzyme) of the XoxF proteins to the periplasmic domain of the sensor histidine kinase MxcQ is essential to regulate the switch was postulated (23).

The type I methanotroph Methylomicrobium buryatense strain 5GB1C is lacking homologues of the aforementioned TCS systems MxcQE and MxbDM (13). In this organism, the REE switch is regulated predominantly by the sensor histidine kinase MxaY (29). Chu and coworkers (29) found that the activation of MxaY in the absence of lanthanum activates transcription of the Ca^2+^-dependent enzyme MxaF by a so-far-unknown response regulator. In addition, the deletion of MxaY was found to almost entirely eliminate the Ln^3+^-mediated transcriptional activation of the Ln^3+^-dependent enzyme XoxF in a partially MxaB-dependent manner that also results in a severe growth defect, both in the presence and absence of Ln^3+^. As an additional layer of complexity, recent studies found that the presence of other metal ions such as copper, which is needed as a cofactor for the membrane-bound or particulate methane monooxygenase (pMMO) in methanotrophs, can significantly impact the REE-mediated switch (21, 22, 30).

In contrast to the increasing knowledge about the regulation of PQQ-MDHs in methylotrophs, the molecular basis underlying such a regulatory switch for PQQ-EDHs in nonmethylotrophic organisms is not established. In the present study, we identify the TCS encoded by PP_2671/PP_2672 (hereinafter referred to as PedS2/PedR2 according to the genetic nomenclature from Arias et al. [31]), consisting of the sensor histidine kinase PedS2 and its cognate LuxR-type transcriptional response regulator PedR2, as an essential regulatory module for the REE-mediated switch of PQQ-EDHs in P. putida KT2440. We provide evidence that the activity of PedS2 in the absence of lanthanides leads to phosphorylation of PedR2 (PedR2^P^), which serves a dual regulatory function. On the one hand, PedR2^P^ acts as a strong transcriptional activator of the *pedE* gene, which is essential to allow growth with 2-phenylethanol in the absence of Ln^3+^. At the same time, PedR2^P^ also functions as a repressor of *pedH*. From our data, we conclude that the presence of Ln^3+^ ions triggers a reduction in PedS2 activity, either by a direct binding of the metal to the periplasmic region of PedS2 or by an uncharacterized indirect interaction. This reduction of PedS2 activity, together with a proposed phosphatase activity of PedS2 under this condition, causes the accumulation of nonphosphorylated PedR2, which over time results in the loss of the regulatory activity of the protein, and facilitates—in concert with the positive-feedback function of PedH in the presence of Ln^3+^ ions—the switch between PedE- and PedH-dependent growth.

## RESULTS

### Identification of the sensor histidine kinase PedS2 as a lanthanide-responsive sensor.

**FIG 1:**
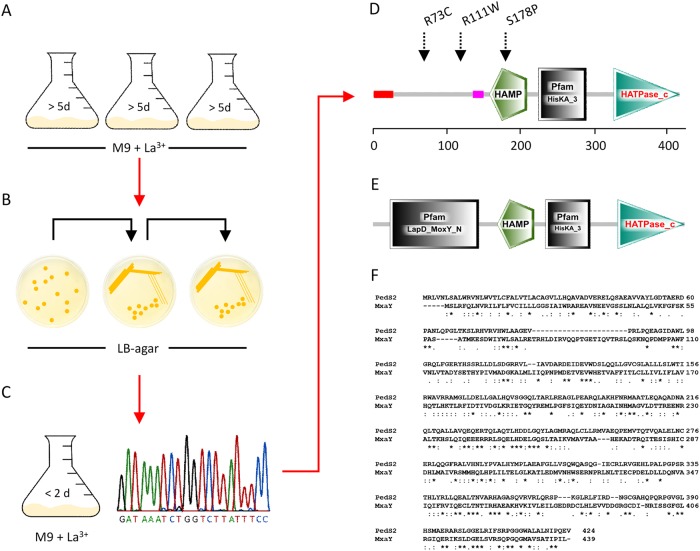
(A to D) Schemes of selection (A), clonal isolation (B), characterization and single nucleotide polymorphism (SNP) identification (C and D) in the two-component sensor histidine kinase PedS2 of the Δ*pedH*^S1^ (R73C), Δ*pedH*^S2^ (R111W), Δ*pedH*^S3^ (S178P) spontaneous mutants. (A) Cells of the Δ*pedH* strain were incubated in M9 medium supplemented with 5 mM 2-phenylethanol and 10 µM LaCl_3_ in plastic Erlenmeyer flasks (*n* = 3) at 30°C with shaking at 180 rpm. (B) After growth was observed (>5 days), dilutions from each culture were plated onto LB agar plates and incubated at 30°C. Individual clones were further streaked on LB agar twice prior to further characterization. (C) Clones were characterized for their growth behavior in M9 medium with 5 mM 2-phenylethanol in the presence of 10 µM LaCl_3_. Subsequently, one clone from each culture exhibiting faster growth than the parental Δ*pedH* strain was used for PCR amplification of the *pedS2* gene and multiple-sequence alignment analysis with the native sequence of the gene from the Pseudomonas Genome Database (52). (D and E) Visualization of domain composition of PedS2 of P. putida (D) and MxaY of Methylomicrobium buryatense 5GB1C (E) using the prediction from the Simple Modular Architecture Research Tool (53). (F) Amino acid sequence alignment of the PedS2 and MxaY proteins generated with Clustal Omega (50).

As a consequence of the inverse regulation of *pedE* and *pedH*, a *pedH* deletion strain does not grow within 48 h with 2-phenylethanol as the sole carbon source in the presence of a critical concentration of La^3+^ in the culture medium (8). To test whether strains can evolve to overcome the repression of *pedE* in the presence of Ln^3+^, an adaptive evolution experiment was performed (see Fig. 1 for a general scheme).

When Δ*pedH* cultures were incubated with 2-phenylethanol in the presence of 10 µM La^3+^ ions for longer than 5 days, growth was observed, indicating the occurrence of adapted strains (data not shown). When independent clones were isolated from such cultures and passaged several times on LB agar medium, their growth phenotype with 2-phenylethanol was much faster (<2 days) than that observed for their Δ*pedH* parental strain and very similar to the growth phenotype of the wild-type strain KT2440. Similar spontaneous suppressor mutants have been reported in methylotrophic organisms such as Methylobacterium extorquens strain AM1, Methylomicrobium buryatense strain 5GB1C, and Methylobacterium aquaticum strain 22A during growth with methanol (13, 16, 29). In M. buryatense, whole-genome sequencing revealed that the suppressor mutant strain was characterized by a mutation in the membrane-bound two-component sensor histidine kinase MxaY (29). In Pseudomonas putida KT2440, the gene *PP*_*2671* (hereinafter referred to as *pedS2*), located in close genomic proximity to *pedE* (*PP*_*2674*), encodes a membrane-bound histidine kinase sharing 25% amino acid sequence identity with MxaY (Fig. 1D to F). To test the hypothesis that mutations in *pedS2* are responsible for the emergence of suppressor phenotypes in the Δ*pedH* mutant strain during growth in the presence of La^3+^, the gene was sequenced in three individually isolated mutants (Δ*pedH*^S1^ to Δ*pedH*^S3^ mutant strains) (Fig. 1A to D). This analysis revealed that all strains contained a mutation in the *pedS2* sequence leading to a nonsynonymous substitution. These mutations were either located within a predicted periplasmic domain of unknown function (R73C [Δ*pedH*^S1^ strain] and R111W [Δ*pedH*^S2^ strain]) or within the HAMP domain (S178P [Δ*pedH*^S3^ strain]), which is responsible for signal transduction from the periplasm into the cytoplasm of the cell (32, 33). To verify that the identified mutations in *pedS2* are the primary cause of the suppressor phenotype, the S178P mutation from the Δ*pedH*^S3^ strain was introduced into the genetic background of the Δ*pedH* strain, resulting in the Δ*pedH_*PedS^S178P^ strain.

**FIG 2:**
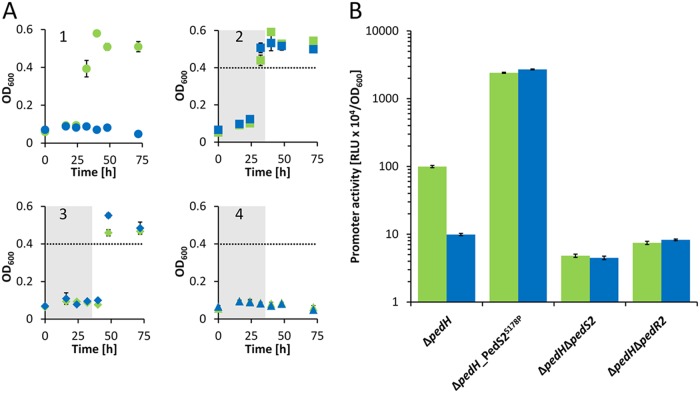
(A) Growth of Δ*pedH*, Δ*pedH*_*pedS2*^S178P^, Δ*pedH* Δ*pedS2*, and Δ*pedH* Δ*pedR2* strains. The Δ*pedH* (circles; panel 1), Δ*pedH*_*pedS2*^S178P^ (squares; panel 2), Δ*pedH* Δ*pedS2* (diamonds; panel 3), and Δ*pedH* Δ*pedR2* (triangles; panel 4) strains were grown at 30°C and 350 rpm shaking with M9 medium in 96-well plates supplemented with 5 mM 2-phenylethanol in the presence of 10 µM La^3+^ (blue symbols) or in the absence of La^3+^ (green symbols). The gray areas in panels 2 to 4 show the time point by which the parental Δ*pedH* strain (circles) reached an OD_600 _of >0.4 (dotted line). (B) Activities of the *pedE* promoter in Δ*pedH*, Δ*pedH*_PedS2^S178P^, Δ*pedH* Δ*pedS2*, and Δ*pedH* Δ*pedR2* strains in the presence (blue bars) of 1 µM La^3+^ or absence of La^3+^ (green bars) or measured in M9 medium supplemented with 1 mM 2-phenylethanol. Promoter activities are presented in relative light units (RLU × 10^4^) normalized to OD_600_. All data represent the means for biological triplicates, and error bars correspond to the respective standard deviations.

In subsequent growth experiments with 2-phenylethanol in the absence of La^3+^, the Δ*pedH* and Δ*pedH_*PedS2^S178P^ mutants showed similar growth behavior with a lag phase of <32 h (Fig. 2A1 and A2). In the presence of 10 µM La^3+^, however, the Δ*pedH* strain showed no growth within 72 h, whereas the Δ*pedH_*PedS2^S178P^ strain reached its maximum optical densities again after about 32 h of incubation, verifying that the observed mutation in the histidine kinase *pedS2* gene was sufficient to cause the suppressor phenotype. We speculated that the LuxR-type response regulator *exaE* (*PP*_*2672*; hereinafter referred to as *pedR2*), which is located adjacent to *pedS2* within the genome of P. putida KT2440, represents the target of PedS2 activity. This assumption is based on the fact that PedR2 represents a homologue (65% amino acid sequence identity) of EraR (ExaE; PA1980), which forms a two-component system (TCS) with the cytosolic histidine kinase EraS (ExaD; PA1979) that activates expression of the *pedE* homologue *exaA* in Pseudomonas aeruginosa (34, 35). To test this hypothesis, *pedS2* as well as its potential cognate response regulator-encoding gene *pedR2*, were deleted in a Δ*pedH* background. In addition, strains suitable for probing promoter activity of *pedE* in Δ*pedH*, Δ*pedH_*PedS2^S178P^, Δ*pedH* Δ*pedS2*, and Δ*pedH* Δ*pedR2* mutant strains were constructed and subsequently analyzed during growth with 2-phenylethanol in the presence and absence of La^3+^ (Fig. 2B).

### The PedS2/PedR2 TCS regulates *pedE* transcription in response to lanthanide availability.

In accordance with the observed growth patterns, *pedE* promoter activities in the Δ*pedH_*PedS2^S178P^ mutant were almost identical in both the presence and absence of La^3+^ (ratio of *pedE* promoter activity in cells grown without La^3+^ to promoter activity in cells grown with 1 µM La^3+^, 0.89 ± 0.02) but increased more than 24-fold (24-fold ± 1-fold and 27-fold ± 1-fold, respectively) compared to the *pedE* promoter activities determined for cells of the Δ*pedH* strain grown in the absence of La^3+^ (Fig. 2B). The Δ*pedH* Δ*pedS2* double mutant also showed a La^3+^-independent growth phenotype similar to that of the Δ*pedH_*PedS2^S178P^ strain but with a clear delay (<48 h versus <32 h) (Fig. 2A3). Promoter activities for *pedE* were almost identical in this strain in the presence and absence of La^3+^ (ratio of *pedE* promoter activity in cells grown without La^3+^ to promoter activity in cells grown with 1 µM La^3+^, 1.07 ± 0.09) (Fig. 2B).

In contrast, incubations of 72 h (Fig. 2A4) or even prolonged incubations for more than 7 days (data not shown) did not result in detectable growth of the Δ*pedH* Δ*pedR2* double mutant with 2-phenylethanol, both in the presence and absence of La^3+^. Correspondingly, *pedE* promoter activities in this strain were low compared to those observed for cells of the Δ*pedH* strain in the presence of La^3+^ but in a range similar to those observed for the Δ*pedH* Δ*pedS2* strain in the presence and absence of La^3+^. These data demonstrate that the PedS2/PedR2 system is the predominant element in the La^3+^-dependent regulation of *pedE* and that PedR2 is essential for PedE-dependent growth. However, as PedE-dependent growth can still be observed in the absence of PedS2 after prolonged incubations and in a PedR2-dependent manner (Fig. 2A3 and A4), we assume that at least one additional lanthanide-independent kinase must be able to phosphorylate PedR2, leading to transcriptional activation of *pedE* and functional production of the calcium-dependent enzyme under these conditions.

### The PedS2/PedR2 TCS regulates the partial repression of *pedH* in the absence of lanthanides.

On the basis of the critical role of PedS2/PedR2 in the regulation of *pedE* and the fact that LuxR-type regulators have been demonstrated to be capable of acting as both transcriptional activators and repressors (36, 37), we speculated that this TCS could also be involved in the regulation of *pedH*. To test this hypothesis, Δ*pedE*_PedS2^S178P^, Δ*pedE* Δ*pedS2*, and Δ*pedE* Δ*pedR2* mutant strains were generated, and a transcriptional reporter suitable for probing *pedH* promoter activities was integrated into the genome of each of these strains.

**FIG 3:**
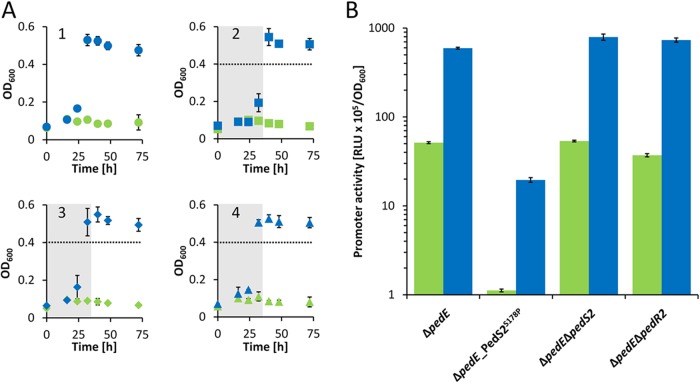
(A) Growth of Δ*pedE*, Δ*pedE*_PedS2^S178P^, Δ*pedE* Δ*pedS2*, and Δ*pedE* Δ*pedR2* strains. The Δ*pedE* (circles; panel 1), Δ*pedE*_PedS2^S178P^ (squares; panel 2), Δ*pedE* Δ*pedS2* (diamonds; panel 3), and Δ*pedE* Δ*pedR2* (triangles; panel 4) were grown at 30°C and 350 rpm shaking with M9 medium in 96-well plates supplemented with 5 mM 2-phenylethanol in the presence of 10 µM La^3+^ (blue symbols) or absence of La^3+^ (green symbols). The gray areas in panels 2 to 4 show the time point by which the parental Δ*pedE* strain (circles) reached their maximum OD_600_. (B) Activities of the *pedH* promoter in Δ*pedE*, Δ*pedE*_PedS2^S178P^, Δ*pedE* Δ*pedS2*, and Δ*pedE* Δ*pedR2* strains in the presence of 1 µM La^3+^ (blue bars) or in the absence of La^3+^ (green bars) or measured in M9 medium supplemented with 1 mM 2-phenylethanol. Promoter activities are presented in relative light units (RLU × 10^5^) normalized to OD_600_. All data represent the means for biological triplicates, and error bars correspond to the respective standard deviations.

**FIG 4:**
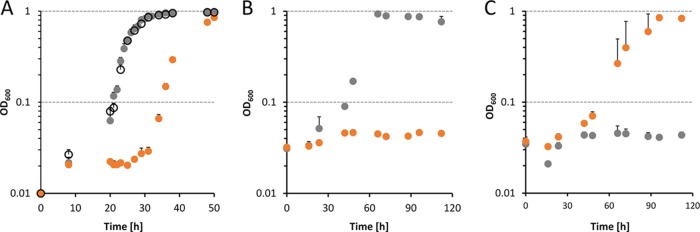
Growth of different P. putida strains at 30°C and 180 rpm shaking with M9 medium in polycarbonate Erlenmeyer flasks supplemented with 5 mM 2-phenylethanol and 10 µM La^3+^ in the absence (A) and presence of kanamycin (B and C) for plasmid maintenance. Flasks were inoculated at an OD_600_ of 0.01 (A) or 0.03 (B and C) with washed cells from M9 overnight cultures grown with succinate in the absence (A) or presence (B and C) of kanamycin and 0.2% (wt/vol) rhamnose to induce pJEM[PedR2] and pJEM[PedR2^D53A^] plasmids. (A) Growth of Δ*pedE* (black circles), Δ*pedE*_PedS2^S178P^ (orange circles), and Δ*pedE*_PedS2^S178P^ Δ*pedR2* (gray circles) strains. (B) Growth of Δ*pedH*_PedS2^S178P^ Δ*pedR2* strain harboring pJEM[PedR2] (gray circles) or pJEM[PedR2^D53A^] (orange circles). (C) Growth of Δ*pedE*_PedS2^S178P^ Δ*pedR2* strain harboring pJEM[PedR2] (gray circles) or pJEM[PedR2^D53A^] (orange circles). Data points represent the means for biological triplets, and error bars correspond to the respective standard deviations (positive error values).

Experiments with 2-phenylethanol revealed that Δ*pedE*, Δ*pedE* Δ*pedS2*, and Δ*pedE* Δ*pedR2* mutant strains showed La^3+^-dependent growth after a lag phase of <24 h and reached the maximum optical density at 600 nm (OD_600_) after ≤32 h (Fig. 3A1, A3, and A4). In contrast, the Δ*pedE*_PedS2^S178P^ strain exhibited an extended lag phase (>24 h) and consistently reached the maximum OD_600_ only after prolonged incubations (≥32 h [Fig. 3A2]). In accordance with these growth results, the *pedH* promoter activities of Δ*pedE*, Δ*pedE* Δ*pedS2*, and Δ*pedE* Δ*pedR2* strains were in a similar range, whereas *pedH* promoter activities in the Δ*pedE*_PedS2^S178P^ strain were 45-fold ± 2-fold and 30-fold ± 2-fold lower than the promoter activities of the Δ*pedE* strain in the absence or presence of La^3+^, respectively (Fig. 3B). Assuming that the S178P mutation in PedS2 results in a sensor kinase activity that mimics that of the wild-type protein in the absence of lanthanides, it is very likely that PedS2 is responsible for the repression of *pedH* under the La^3+^-free conditions leading to the observed delay in growth. To find out whether this regulatory effect on *pedH* proceeds via the response regulator PedR2, as is the case for *pedE*, or is caused by an unknown additional target of PedS2, the Δ*pedE*_PedS2^S178P^ Δ*pedR2* triple mutant strain was generated and characterized for its growth phenotype (Fig. 4A). In this experiment, the Δ*pedE* and Δ*pedE*_PedS2^S178P^ strains both grew with 2-phenylethanol but with clear differences in the corresponding lag phases and maximum growth rates (0.087 ± 0.003 h^−1^ versus 0.057 ± 0.001 h^−1^), confirming the previous results from growth in 96-well plates (Fig. 3A). In contrast, the additional deletion of the response regulator PedR2 eliminated the growth defect caused by the PedS2^S178P^ allele, leading to a growth behavior of the Δ*pedE*_PedS2^S178P^ Δ*pedR2* strain (Fig. 4A), which was indistinguishable (maximum growth rate, 0.089 ± 0.003 h^−1^) from that of the Δ*pedE* strain.

### The conserved phosphorylation site D53 in PedR2 is essential for the REE-mediated switch.

In order to study the essentiality of the phosphorylation site at position D53 of PedR2 (38, 39), we used inducible low-copy-number constructs for the production of the wild-type PedR2 protein (pJEM[PedR2]) and a mutated variant, in which the conserved aspartate in the CheY-like receiver domain was replaced by an alanine (pJEM[PedR2^D53A^]). After transformation of these plasmids into the Δ*pedH* Δ*pedR2* and Δ*pedE*_PedS2^S178P^ Δ*pedR2* mutant strains, their growth with 2-phenylethanol in the presence and absence of La^3+^ was monitored. When the plasmid-borne wild-type regulator PedR2 was induced in cells of the Δ*pedH* Δ*pedR2* strain, growth was observed after a lag phase of <24 h (maximum growth rate, 0.032 ± 0.005 h^−1^), whereas the PedR2^D53A^ variant was unable to restore PedE-dependent growth in the same strain (Fig. 4B). Intriguingly, the reverse result was obtained in the Δ*pedE*_PedS2^S178P^ Δ*pedR2* strain. Here, the PedR2^D53A^ variant allowed PedH-dependent growth (maximum growth rate, 0.026 ± 0.002 h^−1^), whereas the wild-type regulator PedR2 did not lead to significant growth within 120 h of incubation (Fig. 4C).

## DISCUSSION

**FIG 5:**
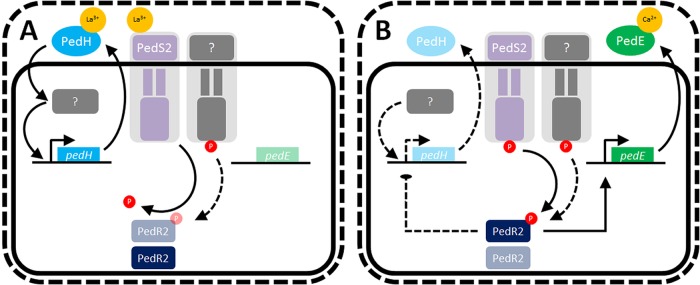
Working hypothesis of rare earth element (REE)-mediated switch of *pedE* and *pedH* in Pseudomonas putida KT2440. (A) The presence of REEs in the medium leads to binding of Ln^3+^ ions to PedH and the periplasmic domain of PedS2. The binding to PedH leads to the catalytic activation of the enzyme and triggers enhanced transcriptional activation of *pedH* by a so-far unknown mechanism and regulator, which is indicated by a gray box (8). Binding to the periplasmic domain of PedS2 leads to an outside-in signaling via the HAMP domain and decreases the kinase activity of the protein toward its cognate response regulator PedR2. In addition, this state of the PedS2 sensor is believed to exhibit phosphatase activity to reduce cross talk of an unidentified kinase (indicated by a gray membrane-bound protein) with activity toward PedR2 (for a more detailed explanation, see the text). (B) In the absence of REEs, the sensor kinase PedS2 is active and phosphorylates the cognate response regulator PedR2 at position D53. The phosphorylated PedR2 has a dual regulatory function as a strong *pedE* activator and a repressor of *pedH*. Black lines indicate known regulations or functionalities. Solid lines indicate a strong regulatory effect on genes or production of enzymes, whereas dotted lines indicate weaker regulatory effects or low production of enzymes.

We recently demonstrated that in P. putida KT2440, the production of the two PQQ-EDHs PedE and PedH is both tightly and inversely regulated depending on lanthanide availability, representing the first reported REE switch for PQQ-EDHs in a nonmethylotrophic organism (8). In this study, we were able to show that Ln^3+^-dependent transcriptional activation of *pedH* is mostly, but not entirely, dependent on the presence of the PedH protein itself by a so-far unknown mechanism and transcriptional regulator (Fig. 5A). Notably, the Ln^3+^-dependent transcriptional repression of *pedE* remained elusive. In the current study, we present a detailed characterization of the mechanism underlying PedE and PedH regulation, in which the PedS2/PedR2 TCS acts as an essential signaling module for the REE-mediated switch between the two quinoproteins.

Similar to the recently characterized spontaneous mutant of M. buryatense (29), we found that a single nonsynonymous mutation within the periplasmic region of the sensor histidine kinase PedS2 (PedS2^S178P^), which differs from the LapD/MoxY domain found in MxaY of M. buryatense (Fig. 1), is sufficient to terminate the Ln^3+^-mediated repression of *pedE*. Notably, various mutations at different sites of the protein can cause the observed suppressor phenotype. This might explain the repeated and fast occurrence of these suppressor mutants in our experiments and would support a similar notion in M. buryatense (13, 29).

Besides the essentiality for *pedE* regulation, our experimental data further provide strong evidence that PedS2 is also involved in the repression of *pedH* in the absence of lanthanides, but not in its Ln^3+^-dependent activation. This is based on the observation that the Δ*pedE* Δ*pedS2* deletion strain did not show any differences in growth and *pedH* activation, while the Δ*pedE*_PedS2^S178P^ suppressor mutant displayed decreased *pedH* promoter activity and a strongly increased lag phase in growth experiments in the presence of La^3+^. The complementation assay with the inducible PedR2 variants further demonstrates that the *pedS2*-dependent regulation of *pedE* and *pedH* is mediated by the LuxR-type response regulator PedR2 for both genes. From these data, we conclude that in the absence of La^3+^ ions, the PedS2 sensor histidine kinase is active and triggers phosphorylation of PedR2 at the conserved position D53 (PedR2^P^) (38, 39). The phosphorylated state of PedS2 subsequently has a dual regulatory function, namely, the activation of *pedE* and concomitant repression of *pedH* transcription (Fig. 5A). In this context, it is interesting to note that expression of the *exaA* gene in Azospirillum brasilense Sp7, which encodes a pyrroloquinoline quinone (PQQ)-dependent alcohol dehydrogenase, is dependent on σ^54^ and its interaction with a LuxR-type response regulator that shares 45% sequence identity with PedR2 (40). Whether the transcriptional activation of *pedE* is dependent on a similar interaction of PedR2 with a specific sigma factor is currently unknown.

To our surprise, a strain lacking PedS2 (Δ*pedH* Δ*pedS2* strain) was still able to grow on 2-phenylethanol in the absence of Ln^3+^, even though it exhibited an increased lag phase and low *pedE* promoter activities (Fig. 2A3). As our study provides strong evidence that phosphorylation of PedR2 is essential for transcriptional activation of *pedE*, this observation indicates that an additional so-far unidentified kinase beside PedS2 is capable of phosphorylating PedR2 to facilitate growth of this strain under these conditions (indicated in Fig. 5 as a gray membrane-bound protein). Given that such an additional kinase exists, it is even more surprising that a *pedH* single mutant, in contrast to the aforementioned Δ*pedH* Δ*pedS2* double mutant, is able to grow in the presence of REEs only when PedS2 is mutated (e.g., PedS2^S178P^ [Fig. 2A1]). This suggests that the activity of the additional kinase toward PedR2 in the presence of La^3+^ is repressed as long as PedS2 is functional. As an intrinsic phosphatase activity has been found for many bacterial sensory histidine kinases (33, 41, 42), we hence propose that PedS2 also exhibits phosphatase activity on PedR2^P^ in the presence of La^3+^, thereby ensuring specificity of the signal transduction pathway and eliminating interference from other nonspecific kinases. In our working hypothesis, the presence of lanthanides in the medium leads to the repression of PedS2 kinase activity, most likely by direct binding of the metal ions to its periplasmic domain. The reduced kinase activity and postulated phosphatase activity of PedS2 in the presence of Ln^3+^ consequently leads to the accumulation of unphosphorylated PedR2, which finally results in the loss of its regulatory functions. In addition, the transcription of *pedH* is activated via a yet unknown pathway, in which a functional PedH protein is an essential component, most likely by acting as a lanthanide sensor (8).

In conclusion, it appears that the REE-mediated switches in Methylobacterium extorquens AM1 and Methylomicrobium buryatense during growth with methanol are predominantly dependent on only one lanthanide-responsive pathway, which either proceeds via the XoxF1 and XoxF2 proteins or via the MxaY protein (23, 29). Our results establish that in P. putida KT2440, a combination of at least two independent pathways are important to orchestrate the inverse regulation of *pedE* and *pedH* in response to lanthanides efficiently.

Several recent studies suggest that in methano- and methylotrophic bacteria, the REE switch might affect more genes than only the genes needed for the periplasmic oxidation system itself (16, 22, 43). Reports on physiological consequences are, however, inconsistent as some studies found no effects (13, 16, 23, 44), whereas other studies reported a stimulating effect on biofilm formation, growth rates, and overall yields in the presence of REE (43, 45). We think it is not unlikely that additional REE-mediated regulatory effects also exist in P. putida KT2440 in a context-dependent manner. Thus, one of our current foci is to investigate the global regulatory impact and physiological consequences of the presence and absence of lanthanides under various environmental conditions.

## MATERIALS AND METHODS

### Bacterial strains, plasmids, and culture conditions.

A detailed description of the bacterial strains, plasmids, and primers used in this study can be found in Tables 1 and 2. If not stated otherwise, Escherichia coli and Pseudomonas putida KT2440 strains were maintained on solidified LB medium. Routinely, strains were cultured in liquid LB medium (46) or a modified M9 salt medium (8) supplemented with 25 mM succinate or 5 mM 2-phenylethanol as a source of carbon and energy at 30°C and shaking, if not stated otherwise. For maintenance and selection, 40 µg ml^−1^ kanamycin or 15 µg ml^−1^ gentamicin for E. coli or 40 µg ml^−1^ kanamycin, 20 µg ml^−1^ 5-fluorouracil, or 15 µg ml^−1^ gentamicin for P. putida strains was added to the medium, if indicated.

**TABLE 1:** Bacterial strains and plasmids used in this study

Bacterial strain or plasmid	Relevant feature(s)	Reference
Bacterial strains		
KT2440	Wild-type strain of Pseudomonas putida (ATCC 47054)	
KT2440*	KT2440 with a markerless deletion of *upp*; parent strain for deletion mutants	48
Δ*pedE*	KT2440* with a markerless deletion of *pedE*	7
Δ*pedE* Δ*pedS2*	Δ*pedE* strain with a markerless deletion of *pedS2* (*PP_2671*)	This study
Δ*pedE* Δ*pedR2*	Δ*pedE* strain with a markerless deletion of *pedR2* (*PP_2672*)	This study
Δ*pedE*_PedS2^S178P^	Δ*pedE* strain with S178P mutation in PedS2	This study
Δ*pedE*_PedS2^S178P^ Δ*pedR2*	Δ*pedE*_PedS2^S178P^ strain with a markerless deletion of* pedR2*	This study
Δ*pedH*	KT2440* with a markerless deletion of *pedH*	7
Δ*pedH* Δ*pedS2*	Δ*pedH* strain with a markerless deletion of *pedS2*	This study
Δ*pedH* Δ*pedR2*	Δ*pedH* strain with a markerless deletion of *pedR2*	This study
Δ*pedH*_PedS2^S178P^	Δ*pedH* strain with S178P mutation PedS2	This study
Δ*pedH*_PedS2^S178P^ Δ*pedR2*	Δ*pedH*_PedS2^S178P^ strain with a markerless deletion of* pedR2*	This study
E. coli BL21(DE3)	F^*−*^* ompT gal dcm lon hsdSB*(r_B_^−^ m_B_^−^) λ(DE3 [*lacI lacUV5-T7* gene* 1 ind1 sam7 nin5*])	
E. coli DH5α	*fhuA2 lac*(del)*U169 phoA glnV44* φ80′ *lacZ*(del)M15* gyrA96 recA1 relA1 endA1 thi-1 hsdR17*	
KT2440*::Tn*7*-*pedE-lux*	KT2440* with insertion of miniTn*7*-*pedE-lux*	8
KT2440*::Tn*7*-*pedH*-*lux*	KT2440* with insertion of miniTn*7*-*pedH*-*lux*	8
Δ*pedE*::Tn*7*-*pedE-lux*	Δ*pedE* strain with insertion of miniTn*7*-*pedE-lux*	8
Δ*pedE*::Tn*7*-*pedH-lux*	Δ*pedE* strain with insertion of miniTn*7*-*pedH-lux*	8
Δ*pedH*::Tn*7*-*pedE-lux*	Δ*pedH* strain with insertion of miniTn*7*-*pedE-lux*	8
Δ*pedH*::Tn*7*-*pedH-lux*	Δ*pedH* strain with insertion of miniTn*7*-*pedH-lux*	8
Δ*pedE* Δ*pedS2*::Tn*7*-*pedH-lux*	Δ*pedE* Δ*pedS2* strain with insertion of miniTn*7*-*pedH-lux*	This study
Δ*pedE* Δ*pedR2*::Tn*7*-*pedH-lux*	Δ*pedE* Δ*pedR2* strain with insertion of miniTn*7*-*pedH-lux*	This study
Δ*pedE*_PedS2^S178P^::Tn*7*-*pedH-lux*	Δ*pedE*_PedS2^S178P^ strain with insertion of miniTn*7*-*pedH-lux*	This study
Δ*pedH* Δ*pedS2*::Tn*7*-*pedE-lux*	Δ*pedH* Δ*pedS2* strain with insertion of miniTn*7*-*pedE-lux*	This study
Δ*pedH* Δ*pedR2*::Tn*7*-*pedE-lux*	Δ*pedH* Δ*pedR2* strain with insertion of miniTn*7*-*pedE-lux*	This study
Δ*pedH*_PedS2^S178P^::Tn*7*-*pedE-lux*	Δ*pedH*_PedS2^S178P^ strain with insertion of miniTn*7*-*pedE-lux*	This study

Plasmids		
pJeM1	Rhamnose-inducible vector (pBBR1MCS backbone) with low copy number	51
pJOE6261.2	Suicide vector for gene deletions	48
pMW55	pJOE6261.2-based deletion vector for gene *PP_2671*	This study
pMW56	pJOE6261.2-based vector for introducing the S178P mutation in PedS2	This study
pMW61	pJOE6261.2-based deletion vector for gene *PP_2672*	This study
pUC18-mini-Tn*7*T-*pedE*-*lux-Gm*	pUC18-mini-Tn*7*T-*Gm*-*lux* vector with the promoter of *pedE* driving transcription of *luxCDABE*	8
pUC18-mini-Tn*7*T-*pedH*-*lux*-*Gm*	pUC18-mini-Tn*7*T-*lux*-*Gm* vector with the promoter of *pedH* driving transcription of *luxCDABE*	8
pTNS2	Helper plasmid for Tn*7* integration	49
pJEM[PedR2]	Rhamnose-inducible induction of PedR2	This study
pJEM[PedR2^D53A^]	Rhamnose-inducible induction of PedR2^D53A^	This study

**TABLE 2:** Primers used in this study

Primer	Primer sequence (5′ → 3′)	Annealing temp (°C)
MWH85	GGAAATATGCAGAAAGTAGCGCTCG	60
MWH86	TCTTCACCACTGGCGGCCT	60
MWH90	GCCGCTTTGGTCCCGCAGGCACTGGCTGCTGC	60
MWH91	CGATATTCAAAGCGGTTCTCCTCAGGC	60
MWH92	GAACCGCTTTGAATATCGTGTTGGTCGATGACCAC	60
MWH93	GCAGGTCGACTCTAGAGGATGCACAAGCTCGGCG	60
MWH98	GCCGCTTTGGTCCCGAGGTAGTAATTCAGTGCGGGGG	60
MWH99	TGCCCGCCTGGGACCTGGTG	60
MWH100	GGTCCCAGGCGGGCAATTG	60
MWH101	GCAGGTCGACTCTAGAGGCAGCCATTGTCGCGAATG	60
MWH106	GCCGCTTTGGTCCCGGCAGGAGCAGGAGCGTAC	65
MWH107	TGAAATACCCACACCTCCTGGGGAATGTTAAG	65
MWH108	GGAGGTGTGGGTATTTCATTGCACCTGTTGGGGC	65
MWH109	GCAGGTCGACTCTAGAGGAGCCAACCTGACCC	65

### Liquid medium growth experiments.

All liquid growth experiments were carried out using modified M9 medium with 25 mM succinate or 5 mM 2-phenylethanol as the sole source of carbon and energy (see above) in 125-ml or 250-ml polycarbonate Erlenmeyer flasks (Corning) or in 96-well 2-ml deep-well plates (Carl Roth) as described previously (8). Briefly, washed cells from overnight cultures grown with succinate at 30°C and 180 rpm shaking were used to inoculate fresh medium with an optical density at 600 nm (OD_600_) of 0.01 and incubated at 30°C and 180 rpm (growth experiments in polycarbonate Erlenmeyer flasks) or 350 rpm (growth experiments in 96-well plates) shaking. Maximum growth rates were calculated from three time points during the exponential phase of growth.

### Construction of plasmids.

For construction of the deletion plasmids pMW55, pMW56, and pMW61, the 600-bp regions upstream and downstream of the *pedS2* gene (*PP_2671*) or amino acid residue S178 in the *pedS2* gene (*PP_2671*) or *pedR2* gene (*PP_2672*) were amplified from genomic DNA of P. putida KT2440 using primers MWH90 to MWH93, MWH98 to MWH101, or MWH106 to MWH109 (Table 2). The two up- and downstream fragments and BamHI-digested pJOE6261.2 were then joined together using one-step isothermal assembly (47). Upon subsequent transformation of the constructs into E. coli BL21(DE3) cells, the correctness of the plasmids was confirmed by Sanger sequencing. Plasmids pJEM[PedR2] and pJEM[PedR2^D53A^] encoding PedR2 or PedR2 with mutated amino acid residue 53 (D→A) under a rhamnose-inducible promoter were ordered from an external source (Eurofins).

### Strain constructions and isolation of suppressor mutants.

The *pedS2* (*PP_2671*) and *pedR2* (*PP_2672*) negative mutants as well as the PedS2^S178P^ allele were constructed using a recently described system for markerless gene deletion in P. putida KT2440 (48). Briefly, the integration vectors harboring the up- and downstream regions of the target genes (pMW55 and pMW61) or the up- and downstream regions of the region to be mutated, including the desired S178P mutation (pMW56) were transformed into P. putida KT2440* and kanamycin-resistant (Kan^r^) and 5-fluorouracil-sensitive (5-FU^s^) clones were selected on LB Kan agarose plates. After incubation at 30°C for 24 h in LB medium without selection markers, clones that were 5-FU resistant (5-FU^r^) and Kan^s^ were tested for successful gene deletion using primer pair MWH90/MWH93 or MWH106/MWH109 for the *pedS2* or *pedR2* gene, respectively. The presence of the underlying PedS2^S178P^ mutation was verified by Sanger sequencing after the *pedS2* gene of 5-FU^r^ and Kan^s^ clones was amplified using primer pair MWH85/MWH86. Δ*pedH* suppressor mutant strains were isolated from 25-ml liquid M9 cultures with 5 mM 2-phenylethanol as the sole source of carbon and energy supplemented with 10 µM La^3+^ upon >5-day incubation at 30°C and 180 rpm. Strains were passaged three times in LB agar plates and reevaluated for growth in liquid M9 medium supplemented with 5 mM 2-phenylethanol and 10 µM La^3+^. From cultures that showed growth with a lag phase similar to that of the wild-type KT2440* strain, the *pedS2* gene was amplified by PCR using primer pair M85/M86, and mutations were identified by Sanger sequencing.

To construct reporter strains for the analysis of *pedE* and *pedH* promoter activity in different genetic backgrounds, plasmids pUC18-mini-Tn*7*T-*pedE*-*lux-Gm* and pUC18-mini-Tn*7*T-*pedH*-*lux-Gm* (8) were coelectroporated with the helper plasmid pTNS2 into selected mutant strains of P. putida KT2440 (Table 1). Proper chromosomal integration of the mini-Tn*7* element in gentamicin-resistant transformants was verified by colony PCR using P_put-glmSDN_ and P_Tn7R_ primers as described previously (49).

### Reporter gene fusion assays.

For quantitative measurement of *pedE* and *pedH* promoter activity, strains of P. putida harboring a Tn*7*-based *pedE-lux* or *pedH-lux* transcriptional reporter fusion were grown overnight in LB medium with gentamicin (15 µg ⋅ ml^−1^), diluted to an OD_600_ of 0.2 in fresh LB medium, and grown to an OD_600_ of 0.6. The cells were then washed three times in M9 medium without a carbon source and finally adjusted to an OD_600_ of 0.2 in M9 medium with 1 mM 2-phenylethanol. For luminescence measurements, 198 µl of a cell suspension was added to 2 µl of a 100 µM LaCl_3_ solution in white 96-well plates with a clear bottom (µClear; Greiner Bio-One). Microtiter plates were placed in a humid box to prevent evaporation and incubated at 28°C with continuous agitation (180 rpm), and light emission and OD_600_ were recorded at regular intervals in an FLX-Xenius plate reader (SAFAS, Monaco) for 6 h. For both parameters, the background provided by the M9 medium was subtracted, and the luminescence was normalized to the corresponding OD_600_. Experiments were performed with biological triplicates, and data are presented as the mean values with error bars representing the corresponding standard deviations.

### Sequence identity determination.

Protein sequence identities were determined based on amino acid sequence alignments of the proteins of interest generated using the Clustal Omega multiple-sequence alignment tool (50).
